# Pultruded Hybrid Reinforced Compounds with Glass/Cellulose Fibers in a Polybutylene Terephthalate Matrix: Property Investigation

**DOI:** 10.3390/polym14061149

**Published:** 2022-03-13

**Authors:** Christian Kahl, André Schlink, Hans-Peter Heim

**Affiliations:** Institute of Material Engineering, Polymer Engineering, University of Kassel, 34125 Kassel, Germany; andre.schlink@uni-kassel.de (A.S.); heim@uni-kassel.de (H.-P.H.)

**Keywords:** hybrid, mechanical properties, CT analysis, pultrusion

## Abstract

The fiber type, orientation of the fiber, fiber-matrix adhesion, and the fiber length are very important for the performance of a short fiber reinforced plastic. Hybrid reinforced polybutylene terephthalate and reference compounds were tested using tensile, Charpy impact, and three-point bending mechanical tests. The interaction of regenerated cellulose fiber and glass fiber was investigated using a polybutylene terephthalate matrix at a fiber volume content of 10%. The ratios of each fiber type was varied. The compounds were pultruded with an extrusion die to have an even fiber length of 3 mm after granulating. In a second step, the specimens were injection molded for mechanical testing. The results were compared to the rule of hybrid mixtures (RoHM) prediction. It was shown that the results of the hybrid reinforced compound were close to the RoHM prediction. The Charpy impact tests show a high positive hybrid effect. The fiber length shows an interaction that is dependent on the ratio of each fiber type.

## 1. Introduction

Thermoplastics reinforced with glass fiber (GF) are used for products where a low weight, low cost, and high thermomechanical properties are required [1]. GF is the most commonly used type of fiber to reinforce thermoplastics [2]. The GF increases the tensile properties of a compound, which, for example, has already been investigated by Miwa, et al. in 1994 [3,4]. Zarges et al. have shown that the critical fiber length for a GF in combination with PP can be influenced by the addition of a coupling agent. This decreases the fiber length due to a stronger bond with the matrix [5]. Man-made cellulose fibers are also used to reinforce a polymer matrix. They are made of regenerated cellulose (RCF) and they are sensitive to high processing temperatures. The low density of 1.5 g/cm^3^ opens up new possibilities for lightweight construction, and the high toughness of the RCF compared to the GF counteracts the brittleness of the composites [6,7]. Due to a very short exposure time of the fibers to a processing temperature above 200 °C, the pultrusion of RCF with, for example, polyamide, was shown in a publication of Feldmann et al. [8,9]. The pultrusion tool used in this publication was also used for the studies in this paper. The processing temperatures of the matrix are comparable here. Reinforcing plastics with two types of fibers opens up new possibilities in the development of the mechanical properties of a composite [10]. A popular combination is RCF and GF. The GF leads to an increase in stiffness and strength, while the RCF increases the toughness of a composite [6,11,12,13]. The interaction of the two fiber types will also be investigated in this publication.

The interaction of the two fiber types GF and RCF has already been investigated in a PP matrix [14]. Since, as described, RCF in particular has a poor bond to the PP matrix, the interaction is to be investigated in this publication using a polar matrix with stronger adhesion. Polybutylene terephthalate (PBT) is an engineering thermoplastic and is widely used for electrical and automotive applications [15]. Gemmeke et al. reinforced PBT with RCF or GF with a weight content of 20 or 30%. They showed that the modulus and the strength increases with an increased fiber content. The GF are shortened significantly due to the process in compounding with an co-rotating double screw extruder [15].

The non-destructive analysis method of X-ray microtomography (µCT) is ideal for the examination of fiber-filled plastic compounds [16,17]. This three-dimensional imaging method enables the characterization of fiber parameters, such as fiber length or fiber orientation. Furthermore, these properties can also be quantified and evaluated in order to relate them to the mechanical properties [14]. The µ-CT was mainly used in this study to evaluate the fiber orientation. Due to the very similar density of RCF and PBT, only the GF can be shown in their length distribution and orientation.

**Figure 1:**
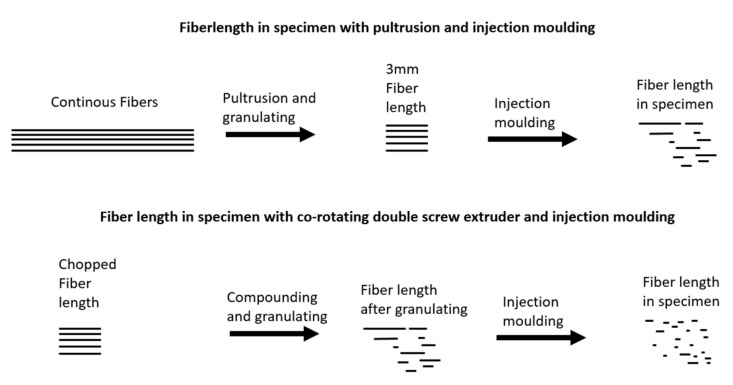
The fiber length in specimens using different compounding techniques.

Compounds produced by pultrusion promise a uniform fiber length after subsequent pelletizing. This fiber length is to be investigated following processing into test specimens by injection molding (Figure 1). In an earlier publication, a strong interface of GF to a PBT was shown compared to the bonding of GF to a polypropylene [18]. In addition to the bonding, the fiber length and the hybrid effect will be investigated by the mechanical characterization at a low fiber volume content of 10 vol%. Hybrid fiber reinforcements with weak fiber-matrix adhesion, such as polypropylenes, are often investigated with high fiber volume contents. The results are compared with the rule of hybrid mixtures (RoHM) and additionally analyzed for fiber lengths and orientation.

## 2. Materials and Methods

### 2.1. Materials

#### 2.1.1. Matrix Polymer

The thermoplastic matrix used in this study is an Ultradur B 4500 PBT (BASF, Ludwigshafen, Germany). The density of this polymer is 1.3 g/cm^3^ and the melt volume rate is 21 cm^3^/10 min at 250 °C/2.16 kg. PBT belongs to the polyester family and has a melting temperature of 223 °C. Before pultrusion and injection molding, the granulate was dried with compressed air at 120 °C for at least 4 h to obtain a moisture content of less than 0.5%. The mechanical properties are shown in Table 1.

#### 2.1.2. Fibers

Rovings made of GF and RCF were used for pultrusion. Data on the density and mechanical properties of the rovings can be found in Table 1. The roving type StarRov 086 is manufactured by Johns Manville and was purchased from Mühlmeier GmbH & Co. KG in Bärnau, Germany. The direct roving made of E-Glass has a soft silane coating on the surface, which promises good bonding to polyesters.

Cordenka CR250 fiber is manufactured by Cordenka GmbH in Obernburg, Germany, using the viscose process. It promises a consistent chemical structure and repeatable properties compared to natural fibers. Since the cellulose regenerated fiber is hydrophilic, the roving was drawn through a chamber heated to 200 °C by a radiator in the pultrusion process. This ensures a low moisture content of the fiber. Prior to injection molding, the compounds were each dried at 120 °C for 4 h.

### 2.2. Experiment

#### 2.2.1. Processing

The fiber length is important for the performance of the compound. In compounding using a twin-screw extruder, the fiber is already heavily shortened. In this study, a continuous fiber is coated by pultrusion and then cut into 3 mm pellets. This provides a uniform fiber length before injection molding, which should result in longer fibers after injection molding into test specimens.

#### 2.2.2. Pultrusion

**Figure 2:**
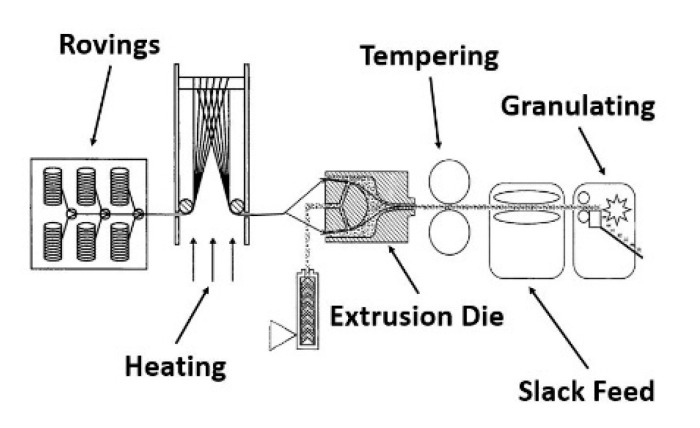
The pultrusion process using an extrusion die [19].

The compounds for this study were produced using a pultrusion process as the first step. A patented tool for impregnating continuous fibers was used in the pultrusion process, according to Figure 2 [19]. The pultrusion speed was set to 0.2 m/s along all compositions. The extruder speed was set to 70 rpm.

The die is connected to a single-screw extruder TR14/24GM from GIMAC (Castronno, Italy). Along the screw are three heating zones where the temperature can be adjusted (Table 2). The rovings are pulled through the die by a slack feed from a spool tree. In the mold, the fibers run over a dome, which is intended to spread them out. From this dome, the melt runs out of the extruder and impregnates the fibers with the melt. At the front of the die, the fibers are recombined and exit a nozzle as an extruded strand. The die is temperature controlled by two heating bands. One heating tape is positioned on the body of the die and another at the nozzle. The pultruded strand passes through rolls of aluminum, which are designed to both compact and cool the strand. Next, the round strand is granulated into 3 mm pieces.

During the pultrusion process, the weight content of the fiber was measured by cutting a meter out of the pultruded strand. The measured weight of the strand was compared to the number of rovings in the process. The weight of the fiber was calculated by the titer and the number of rovings. A variation of the number of rovings and the diameter of the whole in the die were used as the parameters to reach the desired fiber content. The GF and RCF compounds were pultruded separately from each other and mixed in a bag before injection molding into hybrid-reinforced compounds.

#### 2.2.3. Injection Molding

The Arburg Allrounder 320C Golden Edition injection molding machine (Lossburg, Germany) with a clamping force of 50 kN was used to produce the type 1A test specimen (DIN EN ISO 527). All compounds were dried using the TORO-Systems Dry Jet Easy at 120 °C until a moisture content of <0.1 wt% remained. The injection molding machine was equipped with a standard three-section screw with a diameter of 25 mm. Temperatures along the screw ranged from 245 °C to 265 °C. The material was injected at a speed of 24 cm^3^/s, and the back pressure was set high to loosen fiber clusters created by granulating after pultrusion. The mold temperature was 60 °C, and the maximum mold pressure was about 400 bar.

In this study, the fiber content of 10 vol% among all combinations was realized with a variation of the fiber type ratio and the reference compounds with only GF or RCF. The compositions are shown in Table 3.

### 2.3. Characterization

Characterization was carried out on type 1A specimens according to DIN EN ISO 527. The specimens were tested using tensile tests along the longitudinal axis of the specimen. To test the oriented fiber in a different axis, Charpy impact tests and three-point bending tests were carried out. All specimens were conditioned at 23 °C and 50% humidity for at least 24 h before testing. The density of the compounds was measured using the Archimedes method. These values were compared to the calculated density.

The results of the mechanical testing were compared to the RoHM for short fiber reinforced compounds. The comparison shows a negative or positive hybrid effect [20].

(1) $$P_{H}=P_{\mathrm{GF}}*V_{\mathrm{GF}}+P_{\mathrm{RCF}}*V_{\mathrm{RCF}}$$

The RoHM is a formula used to calculate mechanical properties like modulus, toughness, and strength in a composite reinforced with two types of fibers. The property of the hybrid reinforced composite to be investigated is represented by $P_{H}$. This results from the value of the GF compound ($P_{\mathrm{GF}}$) with its volumetric proportion ($V_{\mathrm{GF}}$), as well as the value of the RCF compound ($P_{\mathrm{RCF}}$) and its proportion ($V_{\mathrm{RCF}}$). The formula separates the hybrid into two different systems that have a certain volume in the hybrid reinforced structure.

#### 2.3.1. Tensile Tests

Tensile tests were carried out on a Zwick/Roell Z010 Universal Testing Machine (Ulm, Germany) according to EN ISO 527 on type 1A specimens. The testing speed was set to 5 mm/min. The elongation at break, tensile strength, and tensile modulus were evaluated.

#### 2.3.2. Charpy Impact Tests

To determine the impact strength, instrumented Charpy impact tests were performed according to EN ISO 179 with a Zwick Charpy pendulum (Ulm, Germany) that has a 5 J hammer. Notched specimens measuring 80 × 10 × 4 mm in size were cut out of type 1A specimens for the impact tests.

#### 2.3.3. Three-Point Bending Test

The three-point bending test was carried out according to DIN EN ISO 178 on specimens with the same size as in the Charpy impact test. The tests were also carried out on the Zwick/Roell Z010 Universal Testing Machine (Ulm, Germany). The distance between supports was 64 mm.

#### 2.3.4. Scanning Electron Microscope (SEM)

The crack surfaces of the tensile and Charpy impact tested specimens were observed using an SEM microscope. Their surfaces were coated with gold for application in the ZEISS Ultra-55 Scanning Electron Microscope (Jena, Germany). The images were obtained using magnification ranging from 250× up to 1000×. A magnification of 1000× was carried out to examine the fiber pull-outs and the fiber distribution in the specimens. A magnification of 250× provided an overview of the entire crack surface. The accelerated voltage was 10 kV among all images and an SE detector was used.

#### 2.3.5. Dynamic Image Analysis

For dynamic image analysis, the fibers were extracted from the PBT-matrix of a specimen volume in dichloroacetic acid using an extraction method according to [15]. For the reference samples, one type of fiber was extracted and for the hybrid fiber-reinforced samples, and both types of fiber were extracted from the matrix. The fiber length distribution was measured using a Sympatec QICPIC/R06 (Clausthal-Zellerfeld, Germany) using the MIXCEL wet dispersion unit. Approximately 150,000 fibers were measured for each sample. The parameter LEFI is defined as the shortest distance between the most distant endpoints of a fiber [21]. Images were taken with a frame rate of 175 Hz and a resolution of 4.2 MP using an M4 magnification, which results in a pixel size of 4.2 µm [22].

#### 2.3.6. X-ray Microtomography Analysis

In order to explain the influence of the filler compositions and the specific fiber orientation parameters of GF and RCF composites on the mechanical properties, X-ray microtomography measurements were carried out using an X-ray microscope (Zeiss Xradia Versa 520, Carl Zeiss, Oberkochen, Germany). The results were obtained using a voltage of 80 kV and a current of 87 μA using the low energy filter LE1 and a magnification of 4×. For each three-dimensional measurement, 1601 images were captured with a voxel size of 6.12 μm, binning setting of 2, and an exposure time of 1.6 s for each single image. The reconstruction of the individual images was carried out using Zeiss XMReconstructor software. For further quantitative analysis of the fiber properties and image processing, the 3D Avizo 9.4 data visualization and analysis software system with the XFiber extension (Thermo Fisher Scientific, Waltham, MA, USA) was used. First, a cylinder correlation module was utilized to characterize the fiber orientation in the specimens. Second, to segment the fibers, a tracing algorithm was applied to the resulting correlation lines. The detected fibers can be quantitatively evaluated with regard to their fiber length and orientation in the test specimen. The XFiber extension settings for the cylinder correlation and the tracing algorithm settings are shown in Table 4. To find the correct parameters for fiber tracing, the fiber length distributions of the dynamic image analysis measurements were used as a reference.

The parameter Θ (theta) specifies the fiber orientation. It describes the angle between the z-axis and the xy-plane, and therefore indicates the degree of orientation in the flow direction. A value of Θ = 0° indicates an orientation in the flow direction and a value of Θ = 90° indicates an orientation transverse to the flow direction [23].

The sampling location, which is in the center of the sample to avoid areas of high orientation such as in the boundary layer, is similar to the location in [14].

## 3. Results and Discussion

### 3.1. Density Measurement

Since the density of the fiber type GF and RCF is different, the densities of the hybrid reinforced compound should vary, due to the ratio of the fibers. The results of the density measurement are shown in Table 4. As the GF has a higher density (2.5 g/cm^3^) than the RCF (1.5 g/cm^3^), the density of the compound should increase with the ratio of the GF. The table shows the highest measured density of hybrid reinforced compounds with a high content of GF. A decreasing content of GF leads to lower densities. The measured results are compared to the calculated results and show few deviations from the calculated densities (Table 5).

### 3.2. Mechanical Properties

**Figure 3:**
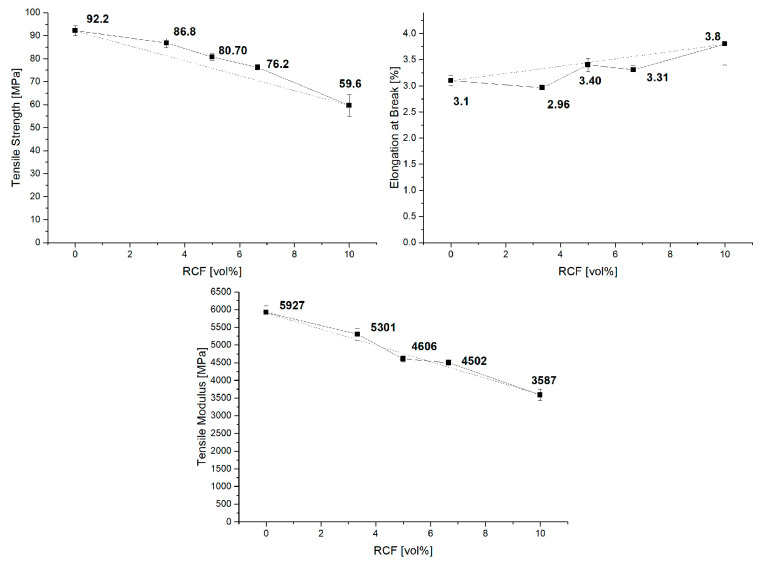
The tensile properties of reference and hybrid reinforced compounds.

The substitution of GF by RCF changes the properties of the compounds. Figure 3 shows the results of the tensile test. The contents of GF and RCF were varied with a constant total fiber volume content. Reference batches with only GF or RCF were also considered. In the graphs, the proportion of RCF is plotted on the horizontal axis. The 0 vol% RCF shows the reference with only GF and 10 vol%; therefore, the reference with only RCF. The hybrid reinforced compounds are shown in between. The dashed line in the diagrams shows a prediction of the results with the RoHM. The reference results show good agreement with the results from the publication by Gemmeke et al. In this publication, GF was produced with a weight fraction of 30%, and a tensile strength of 110 MPa was achieved. Here, the volume content of the GF was set to 10%, which corresponds to a weight fraction of 18%; accordingly, the tensile strength is also lower. A tensile strength of 92 MPa was achieved. The tensile strength of the RCF reinforced PBT also corresponds to this publication. In this publication, the weight percentage of RCF in the reference corresponds to 12%, and the tensile strength is therefore correspondingly low (60 MPa). In the publication by Gemmeke et al., 20 and 30 wt%, respectively, led to strengths of approx. 80 MPa.

The results of the reference compounds show higher values for the Young’s modulus and strength for the GF reference batch due to the stiffer and stronger GF compared to the RCF. Thus, the results of the hybrid reinforced composites should be between the value of the GF and RCF reference batches. For the Young’s modulus, the results of the hybrid compounds show only a slight deviation from the RoHM.

As shown in several publications, RCF compounds have a high deviation compared to GF compounds. The RCF reference compounds show a high standard deviation in strength and elongation at break. In the case of the strength, a clear decrease in the standard deviation can be seen due to the addition of GF. These high standard deviations in the RCF compared to the GF have already been shown in previous publications [14].

The behavior of the tensile properties with a polypropylene matrix was also shown in a previous publication. With polypropylene, poor bonding of the fiber to the matrix is to be expected [18]. A coupling agent was used, which resulted in higher strength in the hybrid reinforced compounds [14]. It was shown that the strength at 16 vol% GF and RCF in different ratios in a PP matrix are also well predictable and close to RoHM.

A different behavior can be seen in the elongation at break. All hybrid reinforced compounds show a negative hybrid effect. This effect can be compared with GF/RCF reinforced PP presented in a different publication. In a PP matrix, the elongation at break increases from 2% to 3.5% when a high amount of GF is substituted by RCF. This result shows a high negative effect. The results of the hybrid reinforced compounds show values that are closer to the prediction of the RoHM [11,14].

**Figure 4:**
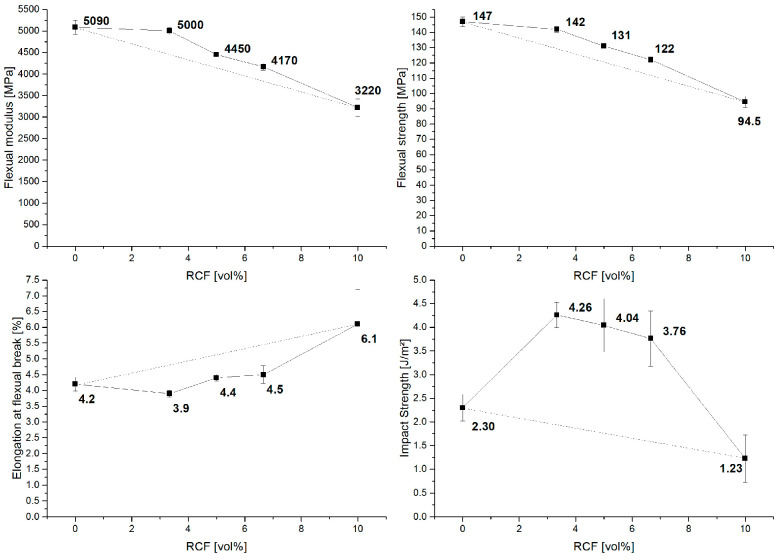
The results of the flexural and notched impact tests with RoHM (dashed line).

The results of the three-point bending (Figure 4) test show a course similar to that of the tensile tests. A positive hybrid effect, with results close to the RoHM, can be seen for stiffness and strength. For the maximum bending strain, the results of the hybrid fiber reinforced compounds are below or close to the reference batch with GF only.

In the Charpy impact tests, the compounds with two fiber types show a strong positive hybrid effect compared to the reference batches with only GF or RCF. However, in this characterization method, the standard deviation is also very high compared to the results of the tensile or three-point bending tests. For the impact results, an increase in the standard deviation with an increasing amount of RCF can be seen.

### 3.3. RoHM

The results of the mechanical characterization were compared with the prediction of the RoHM and it could be observed that in the tensile tests, the deviation from the RoHM is very small. For strength and stiffness, mainly positive hybrid effects can be seen. Negative effects can only be seen in the elongation at break, which decrease up to −12% of the RoHM. For the load transverse to the injection direction by the three-point bending test, the results are similar to the tensile test results. Again, the hybrid effect is positive for stiffness and strength, with 10% for stiffness and high GF content. The maximum strain, on the other hand, stands out with a high negative hybrid effect of up to −24%.

When the specimen is loaded transversely to the flow direction with high velocity, positive hybrid effects with more than 50% can be seen. All hybrid effects are shown in Table 6.

### 3.4. Scanning Electron Microscope (SEM)

**Figure 5:**
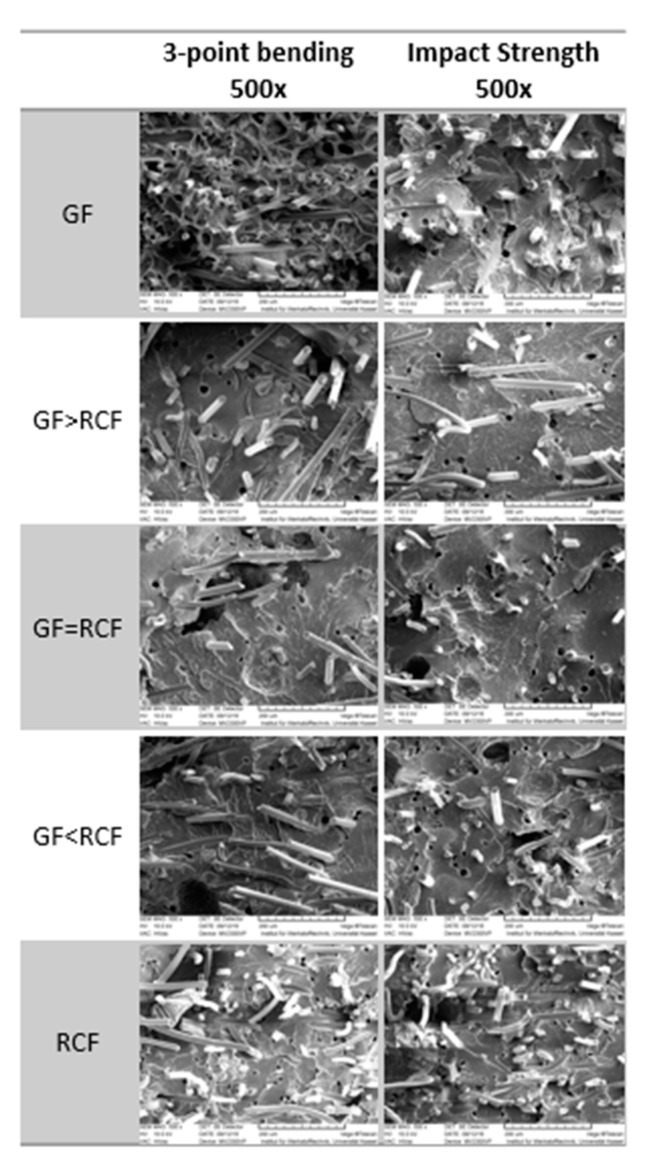
SEM images of broken specimen from flexural and notched impact testing at 500× magnification.

As was shown in other publications, the compound behavior is reflected in the fracture zone, and can be evaluated by SEM [9,24,25]. By adding a high content of RCF, a higher grade of fracture strain and absorbed energy was achieved in impact testing. In this case, the fiber pull-out can be seen in the fracture zone. In contrast, the modulus increases due to a rising GF content, while the strength stays about the same. A high fiber strength is only on hand if the fiber cracks and no pull-outs result. Figure 5 provides a close look at the surface of the fracture zone after the impact testing and the three-point bending test, using a magnification of 500×. The images of reference for GF composition show short broken fibers above the surface. Very few long fiber pull-outs are shown, which results in low deformation and impact results. The hybrid reinforced compositions show more long pulled-out fibers than the reference composites. This results in higher impact strength for the hybrid compositions. Beside the adhesion and the fiber length, the RCF shows a flexural behavior and is not straight oriented, as is the GF. Due to the flexibility and different properties of the fiber, pull-outs of the RCF are expected in a lower number than the GF. The following figure shows long fiber pull-outs in GF.

### 3.5. Dynamic Image Analysis

**Figure 6:**
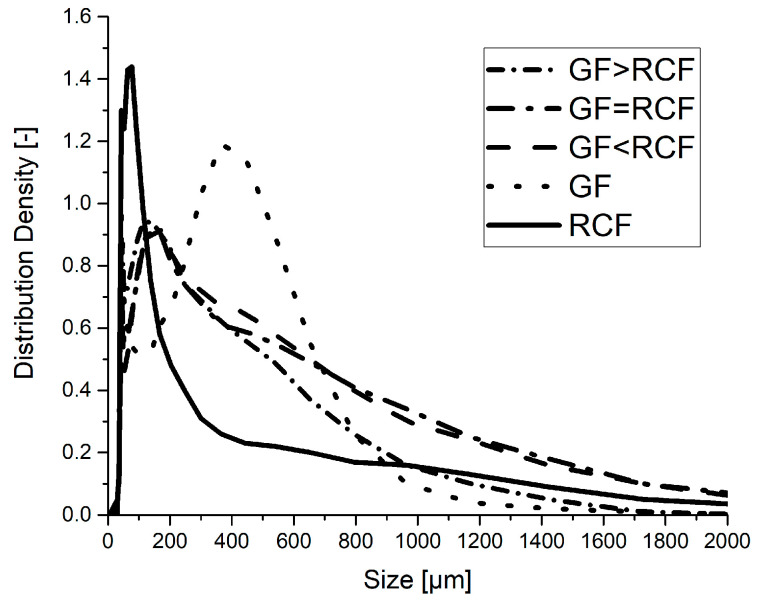
The fiber length distribution measured using dynamic image analysis.

DIA was carried out on the hybrid and reference compounds. The results show the fiber length of RCF and GF in each composition. Figure 6 shows that the reference RCF compound includes only very short fibers, with a high distribution density at about 80 µm. The reference with only GF has the highest distribution density at 400 µm, with a wider range in the peak. The hybrid reinforced compositions have a peak close to the size of the RCF reference (~150 µm). It can be detected that the distribution density of fibers with a length above 700 µm is high in the hybrid reinforced compounds, with a high RCF content. The RCF reference compounds and the hybrid compound with a high GF content show fewer fibers with a length above 700 µm. The RCF thus interacts with the GF during hybrid reinforcement in that the RCF’s flexible appearance helps to protect the fibers during injection molding.

The granules produced after pultrusion have a length of 3 mm. Due to the continuous fiber in pultrusion, the fiber length before the production of test specimens by injection molding can therefore also be considered constant at 3 mm. The severe shortening of the fibers is due to the injection molding process. The high injection pressure was set to dissolve fiber agglomerates through granulation. The result of the high shear forces is very short RCF fibers in the RCF reference sample. These short RCF fibers lead to a low impact Charpy strength. The massive shortening of the RCF counteracts the properties of the RCF. The toughness of the RCF cannot be fully utilized due to the severe shortening.

### 3.6. X-ray Microtomography

**Figure 7:**
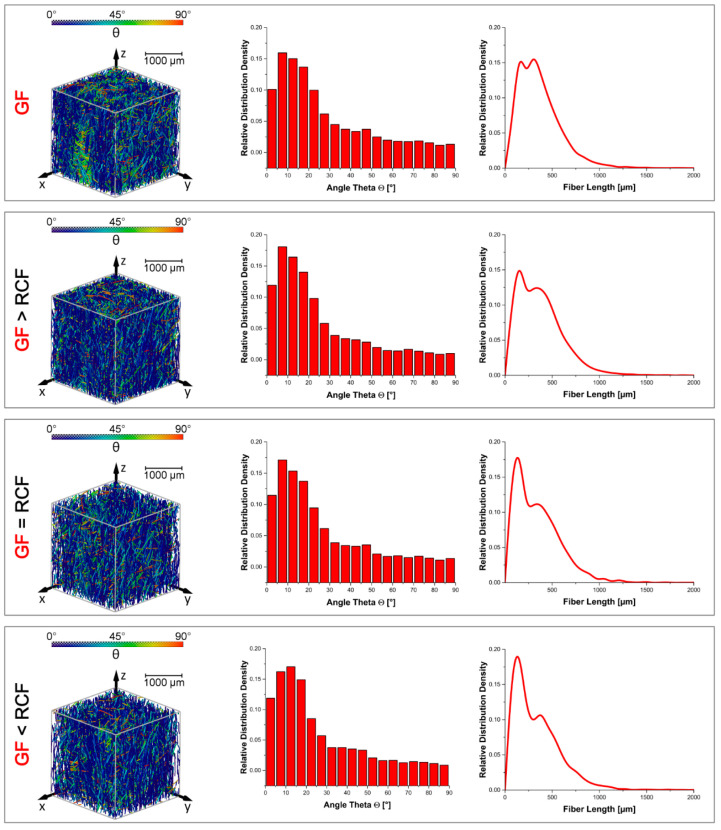
The results of the X-ray microtomography analysis according fiber length and orientation.

Fiber length and fiber orientation were observed using X-ray microtomography. The fiber length in this method shows only the GF. RCF could not be shown in this method because of the very similar densities of the RCF and the PBT. Figure 7 shows the results of the microtomography. The left column shows the oriented fibers in a volume of the specimen as detailed in the publication [14]. The angle θ represents the orientation along the axis in which the fiber is stressed during the tensile test. The smaller the θ is, the better the fiber is oriented. It must be mentioned that clusters appear in the specimen due to the granulated pultruded fiber strand. These clusters can have a negative effect on mechanical performance [26]. The GF reference shows one of these clusters on the surface, with green colored fibers showing an orientation of about 45°. Other volumes of hybrid reinforced compounds exhibit widely well orientated fibers, shown in blue. The bar graph shows the highest distribution density at GF > RCF and GF = RCF, with an angle of θ between 5 and 10°, which is a result of the interaction of the two fiber types in the injection molding process.

The fiber length distribution density of the GF is shown in the right column. The distribution shows two peaks of the GF fiber length; one peak at about 200 µm and the other peak at 400 µm. It can be observed that the peak of 200 µm increases with an increasing RCF content. The second peak at 400 µm therefore decreases in distribution. This prompts the conclusion that the increasing content of RCF leads to a shorter GF and a higher distribution at 200 µm. The DIA results shown in Figure 6 indicate a high distribution density for the hybrid fiber-reinforced compounds at a fiber length of 200 µm. After that, the curves decrease more slowly than for the reference compounds. The computed tomography results suggest that the high distribution density of around 200 µm in the DIA results is created by a large fraction of GF. The strong shortening of the RCF in the reference sample is largely avoided by the interaction of the fibers.

## 4. Conclusions

The study shows the mechanical properties of short fiber reinforced PBT with RCF and GF. A PBT matrix was chosen to have a strong fiber-matrix bonding. The fiber volume content among all compounds was set to 10 vol%, with a variation of the RCF and GF ratio. The fiber length was observed with DIA to show the distribution of both fiber types. In addition, X-ray microtomography was carried out on a volume of the specimens to show the fiber length and the orientation of the GF. According to the results, the following conclusions could be drawn:

- A strong fiber matrix interface was realized with the PBT matrix and was investigated with a small amount of fiber volume (10 vol%) to have observe the interaction of the fiber types. The results of the tensile testing show values close to the RoHM predictions for strength and modulus, with a small positive hybrid effect. The elongation at break shows a negative hybrid effect.
- The three-point bending test shows results similar to the tensile test. The strength and modulus are slightly positive in the hybrid effect, and the elongation at the flexural break shows a negative hybrid effect. The Charpy impact tests show a high positive hybrid effect for the reinforced compounds with two fiber types. The SEM images show broken fibers at the breaking surface for the reference compounds and more long pulled-out fibers for the hybrid compounds. This shows an interaction of the fiber types at a strong interface.
- The DIA results shows that a high amount of the RCF in the reference compound shortens the length to about 100 µm. The GF reference has a high distribution density at 400 µm, which means that the GF appears longer than the RCF. The hybrid compounds show the highest distribution density at about 200 µm. The compounds with higher RCF ratios show a high distribution of longer fibers >800 µm.
- The compounds with a high GF ratio show an increasing distribution density at a small angle θ, which means that the fibers in this compound are well oriented. The GF fiber length measured using X-ray microtomography shows similar results compared to the results of the DIA. The reference GF shows the highest distribution at 400 µm. With an increasing amount of RCF in the hybrid compounds, the GF distribution with a short length of about 200 µm also increases.
- The granulation of the pultruded strand leads to fiber clusters in the specimen. A high injection speed and a high back pressure to loosen fiber clusters leads to short fibers in the reference RCF compounds. These short fibers lead to a low impact strength in the notched Charpy impact test.

## Figures and Tables

**Table 1:** Properties of rovings and PBT matrix [4,15,18].

Roving Type	Fiber Diameter [µm]	Number of Fibers	Density of Material [g/cm^3^]	Density Tex [g/km]	Young’s Modulus [MPa]	Tensile Strength [MPa]	Elongation at Break [%]
E-glass fiber	15.5	1250	2.54	600		3400	3.5
Cordenka Cellulose CR-Type	12	1350	1.5	244	2200	825	13
BASF Ultradur B4500	-	-	1.3	-	2500	55	3.7

**Table 2:** The temperature set on the extruder and the extrusion die.

	Zone 1	Zone 2	Zone 3
Temperature Extruder [°C]	235	245	260
	Tool Body	Nozzle	
Temperature Extrusion Die [°C]	250	260	

**Table 3:** The compositions of reference and hybrid reinforced compounds.

Composition	Weight% GF	Weight% RCF	vol% GF	vol% RCF	vol% Total
GF	18	-	10	-	10
GF > RCF	12	4	6.66	3.3	10
GF = RCF	9	6	5	5	10
GF < RCF	6	8	3.3	6.6	10
RCF	-	12	-	10	10

**Table 4:** The parameters of the fiber tracing analysis of the computer tomography images.

Cylinder length	[μm]	38
Angular sampling		5
Mask cylinder radius	[μm]	10
Outer cylinder radius	[μm]	6
Minimum seed correlation		185
Minimum continuation quality		85
Direction coefficient		0.2
Minimum distance	[μm]	6
Minimum length	[μm]	38

**Table 5:** The measured and calculated densities of the compounds and references at 10 vol%.

Composition	Calculated Density [g/cm^3^]	Measured Density [g/cm^3^]	Difference to Calc. Density [%]
GF	1.43	1.422	−0.56
GF > RCF	1.344	1.38	2.68
GF = RCF	1.335	1.356	1.57
GF < RCF	1.327	1.345	1.36
RCF	1.32	1.315	−0.38

**Table 6:** The mechanical properties results as compared to the RoHM prediction (hybrid effect).

	Tensile			Impact	Three-Point Bending		
	Strength	Modulus	Strain	Strength	Strength	Modulus	Strain
GF < Cell	6.3%	2.9%	−12.5%	54.4%	8.8%	10.7%	−23.9%
GF = Cell	6%	−3.3%	−1.5%	56.3%	7.8%	6.6%	−17.1%
GF > Cell	7.5%	3%	−7.9%	57.7%	8.2%	7.8%	−21.5%

## Data Availability

Not applicable.
